# Development of Analytical Modules of the Geographic Information System Software Complex “Epidemiological Atlas of Russia. Territory of the Federal District” for Additional Analysis of Epidemiological Processes

**DOI:** 10.17691/stm2024.16.6.02

**Published:** 2024-12-27

**Authors:** S.A. Sarskov, M.V. Vyushkov, S.L. Slavin, N.N. Zaitseva

**Affiliations:** Researcher, Laboratory of GIS Technologies and Bioinformatics; Academician I.N. Blokhina Nizhny Novgorod Scientific Research Institute of Epidemiology and Microbiology of Rospotrebnsdzor (Russian Federal Consumer Rights Protection and Human Health Control Service), 71 Malaya Yamskaya St., Nizhny Novgorod, 603950, R; Researcher, Laboratory of GIS Technologies and Bioinformatics; Academician I.N. Blokhina Nizhny Novgorod Scientific Research Institute of Epidemiology and Microbiology of Rospotrebnsdzor (Russian Federal Consumer Rights Protection and Human Health Control Service), 71 Malaya Yamskaya St., Nizhny Novgorod, 603950, R; Student, Institute of Clinical Medicine; National Research Lobachevsky State University of Nizhny Novgorod, 23 Prospekt Gagarina, Nizhny Novgorod, 603022, Russia; MD, DSc, Director; Academician I.N. Blokhina Nizhny Novgorod Scientific Research Institute of Epidemiology and Microbiology of Rospotrebnsdzor (Russian Federal Consumer Rights Protection and Human Health Control Service), 71 Malaya Yamskaya St., Nizhny Novgorod, 603950, R

**Keywords:** geographic information systems in epidemiology, geoinformation software complex, epidemiological monitoring, trends in epidemiological processes

## Abstract

**The aim of the study** is to develop additional analytical modules of geoinformation software complex on current infectious and parasitic diseases aimed to improve the quality of epidemiological monitoring and to generate a database on the trends of epidemical process development in the subjects of the Russian Federation.

**Materials and Methods:**

Additional analytical blocks on comparative and dynamic analysis of morbidity by the groups of nosologies were developed using the software meeting the general concept of the software complex (JavaScript, PHP, and others) and integrated into a new version of the Web application “Epidemiological Atlas of Russia. Territory of the Federal District”. The initial data including information by the groups of diseases were converted into a set of interrelated tables with their further integration into the database of a new version of the Atlas under the control of a free relational MySQL database management system.

**Results:**

The existing classifications of nosologic forms and the search for additional characteristics, potentially forming the groups of nosologies, have been analyzed and the current database of the Epidemiological Atlas has been optimized. The algorithms for obtaining and evaluating epidemiological indicators in the new analytical blocks for estimating cumulative morbidity by the nosologic groups were designed. There were created original analytical modules “Comparative analysis of morbidity by the groups of nosologies” and “Dynamic analysis of morbidity by the groups of nosologies” for the Web application “Epidemiological Atlas of Russia. Territory of Federal District” for the comparative and dynamic morbidity analysis based on the groups of nosologies in the administrative-territorial subject units, in the district subjects, and in the district as a whole, with the possibility of information detailing. The materials based on the database queries contain temporal (calendar month) and spatial detailing (administrative-territory unit of the Russian Federation subject). All materials may be exported as tables, graphs, or maps in various formats (.xls, .pdf, .csv, .png, .jpeg, .svg). Since the databases of the current epidemiological atlases of the Volga Federal District and Russia are universal, the mechanisms of processing tables and queries are identical providing the possibility of using the developed approaches employed in the Epidemiological Atlas of Russia or atlases of other federal districts in case of replicating a new Web application version. New analytical blocks may extend notions on the incidence of current infectious diseases and reveal characteristic regional features, facilitate more exact scientifically grounded proposals for decision-making by the executive authorities and timely taking preventive and anti-epidemic measures.

**Conclusion:**

The developed analytical modules integrated into the new version of the “Epidemiological Atlas of Russia, Territory of the Federal District” were designed to extend the analytical capabilities of the geoinformation software complex. They are characterized by a high significance in optimization and quality improvement of epidemiological monitoring, operative and retrospective epidemiological analysis of current infectious and parasitic diseases in a separate subject, a federal district, and the Russian Federation as a whole, and represent an essential potential for further improvement of analytical methods and technologies.

## Introduction

Infectious diseases are one of the main causes of temporary disability in the population [1]. Geographical information systems (GIS) are a universal tool for the specialists of medical settings, healthcare authorities, and other organizations engaged in the problems of prevention of infectious and non-infectious diseases. Usage of GIS has expanded the capabilities of epidemic process exploration, simplified the analysis of various factors influencing the emergence and spread of diseases among the population [2]. Improvement of the approaches to the evaluation of an epidemic situation is a promising scientific direction. Its development facilitates the scientifically grounded relevant preventive and anti-epidemic activities to reduce or minimize the economic damage from these diseases.

One way to improve the analysis of the epidemic situation on the examined territory in the system of epidemiological surveillance is clustering infections by a common feature, assigning additional characteristics to the studied nosologies in order to identify the trends of epidemic process development for current infectious diseases and to make epidemiological monitoring more effective. The works on the analysis and visualization of infections grouped by a common feature are implemented only in the form of thematic atlases both in Russia and abroad [3–5], they are not geoinformation projects with advanced analytical and interactive tools, their content is not updated for a long time, and they remain the cartographic works relevant for a specific date only.

**The aim of the study** is to develop additional analytical modules of the geoinformation software complex on current infectious and parasitic diseases in the “Epidemiological Atlas of Russia. Territory of Federal District” to improve the quality of epidemiological monitoring and to create a database on the trends of epidemical process development in the subjects of the Russian Federation.

## Materials and Methods

To achieve the goal, the following tasks were being solved:

analysis and preparation of the existing classifications of nosologic forms;
search for additional characteristics potentially forming groups of nosologies;
optimization of the present database “Epidemiological Atlas of Russia. Territory of the Federal District”;
design, development, and integration of the additional analytical modules of the Atlas Web application for comparative and dynamic analysis of morbidity by the groups of nosologies;
development of algorithms for obtaining and evaluating epidemiological indicators in the new analytical blocks.

The analysis of the literature data [6–9] and functioning regulatory documents [10–13] allowed us to form 14 groups and 76 subgroups of infectious diseases (nosologies). The criteria for selecting the characteristics were the possibility to perform an additional analysis across the formed group or subgroup, which would specify the trends of epidemiological processes. The initial data, including the information on the formed groups and subgroups of diseases, were converted to a set of interrelated tables with its subsequent integration into the relational database “Epidemiological Atlas of Russia. Territory of the Federal District”, which allows the user to normalize the work of SQL queries of the existing analytical GIS modules.

The following groups were included into the database: etiological feature, ecological feature, transmission mechanism, main transmission factor, risk factors, preventive measures, epidemiological monitoring, natural nidality, pathogenicity groups, socially significant and dangerous infections, especially dangerous infections, sanitary protection of the territory, ICD-10, immunization schedules. The number of the formed groups is not final and may be changed if necessary (detailing, changes in the groups/subgroups or addition of the new ones). To generate a set of the statistical output values of epidemiological nature in the new analytical blocks, we used information from the previously generated main database of this Atlas, which included data on the subjects and nosologies, tables with the size of population in the municipal units and subjects, data on infectious and parasitic morbidity (forms of Federal Statistical Observation No.1 and No.2) [14].

The morbidity analysis by the groups of nosologies was realized in the form of two supplementary analytical modules: “Comparative analysis of morbidity by the groups of nosologies” and “Dynamic analysis of morbidity by the groups of nosologies”. These modules were formed using the information processing software meeting a general concept of the software complex and previously selected optimal technical solutions (JavaScript, PHP, etc.) [15]; the services and visualization correspond to the previously designed Atlas modules for user convenience in generating queries, working with maps, tables and graphic data on the Web application page. To calculate cumulative morbidity by the groups of nosologies in the new analytical blocks, algorithms of obtaining and evaluating epidemiological indicators have been optimized.

The present work does not contain information that violates anyone’s confidentiality. The study was carried out without the involvement of humans and animals.

## Results

Presently, according to the concept of the software complex, the developed analytical blocks perform the following three tasks: generation of maps, tables, and graphic materials based on the database queries; analysis of epidemiological data; export of tabular and graphic data in the widely used formats (.xls, .pdf, .csv, .png, .jpeg, .svg). Like in the previous blocks, provision is also made for the work with tabular and graphic information and maps (changes of the table content, sorting by the selected attribute indicator, changes in the content of the charts, graphs, and maps). Materials requested from the database are dynamically updated and are formed each time based on the appropriate user query.

**Figure 1.**
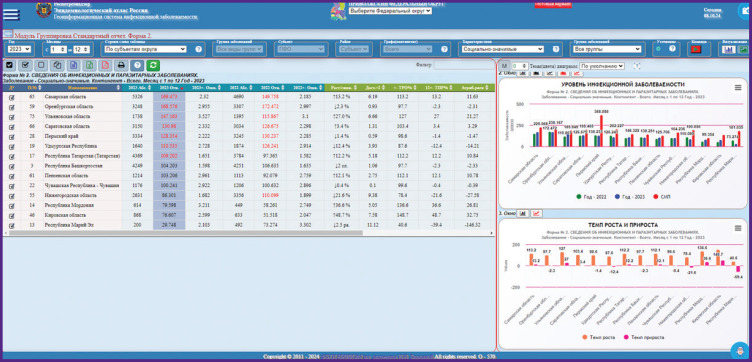
Example of working with the Epidemiological Atlas on the query “Incidence of socially significant infections in the subjects of the Volga Federal District in 2023, °/_°°°°_” using the module “Comparative analysis of morbidity by the groups of nosologies”

***Analytical module “Comparative analysis of morbidity by the groups of nosologies”*** allows the user to make comparative analysis by nosologies, groups of nosologies, periods, territories, and age categories (Figure 1). The selected period (a year, month, months) is compared to the similar period of the previous calendar year. Spatial detailing represents the administrative-territory unit of the district subject (region). Age categories are presented in compliance with Federal Statistic Observation form No.2. The page interface has two main zones: tabular and graphic (maps) presentation of the information.

The tabular result of the query gives the following information: absolute number of cases, relative indications, morbidity rate with error calculation relative to the indicator of the selected periods; comparative analytics of the selected periods (increase rate, growth rate, growth/reduction of morbidity with estimation of reliability, attributable risk, association coefficient); comparative analytics with multi-year average indicators (growth/reduction, reliability) and the line weight in the generated query. To review nosologies, included into the groups and subgroups, detailing and intragroup analysis may be performed.

The maps show the sets of spatially referenced epidemiological information.

**Figure 2.**
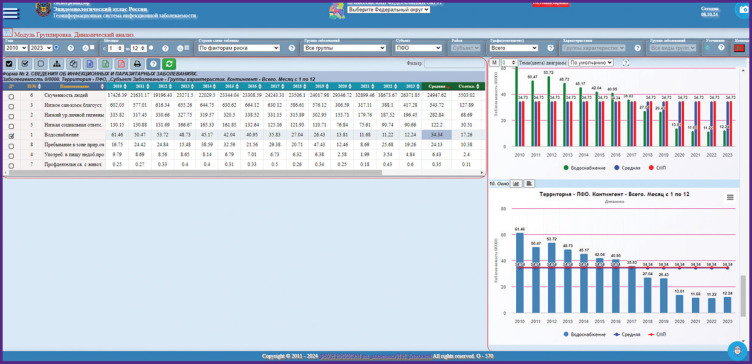
Example of working with the Epidemiological Atlas on the query “Morbidity by the risk factors in the Volga Federal District in 2013–2023, °/_°°°°_” using the module “Dynamic analysis of morbidity by the groups of nosologies”

***Analytical module “Dynamic analysis of morbidity by the groups of nosologies”*** is designed for an in-depth retrospective epidemiological analysis of infectious and parasitic diseases (Figure 2). It helps analyze intra-annual and long-term dynamics in the groups/subgroups of infectious diseases in the administrative-territory units (regions) of a district subject and the entire district; age groups with estimation of multi-year average indicators; indicators of the selected period with estimation of the standard error of relative values; and a short-term morbidity prognosis using linear regression. Detailing and intragroup analysis is also possible for reviewing nosologies included in the groups or subgroups of infectious diseases and/or the age groups.

The distinctive feature of the new analytic blocks is the exclusion function developed for the first time. For example, the share of acute upper respiratory tract infections and a new coronavirus infection (COVID-19) occupies over 90% in the general morbidity structure in all examined subjects, due to which there is a shift in the estimated values and predominance of groups containing these pathologies. The user may exclude acute infections of the upper respiratory tract or COVID-19-associated conditions, or both pathologies simultaneously from the analysis for calculation of multi-year average indicators, the weight in the group structure, building graphs, charts, etc.

## Discussion

Presently, GIS are used by various specialists working at the intersection of informatics, epidemiology, medical statistics, geography, demography, and other sciences. Each year, the number of scientific works on sanitaryepidemiological surveillance using GIS technologies increases in Russia [15–18] and abroad [19–21]. GIS are employed for making strategic decisions both at the level of the territory administration of a separate town and on a nation-wide scale [22].

Taking into account the current state-of-theart and the role of geoinformation technologies in epidemiology, three lines of further research in this field may be outlined: 1) improving the system of information acquisition, the structure and content of the database, increasing initial data reliability; 2) improving the performance characteristics of epidemiologic GIS including a user interface, tools and methods of epidemiological data visualization; 3) improving analytical methods and technologies.

Today, there are no implemented projects on presentation, analysis of group morbidity covering all current infectious and parasitic diseases. One of the ways of improving approaches to the evaluation of an epidemiological situation is clustering infections by a common feature and formation of the groups of nosologies. It makes it possible to increase the efficiency of the epidemiological monitoring owing to the identification of specific regional features characteristic of the current epidemic diseases at all administrative levels. Besides, this method helps define the character of the following groups: 1) by current infectious diseases (especially dangerous, socially significant, and infections, which may lead to emergency situations in terms of sanitary and epidemiological well-being of the population); 2) by the risk factors (crowdness, a long-term contact, violation of indoor conditions such as temperature, airing, humidity; unfavorable sanitary and communal amenity of the territory; a low level of personal hygiene; professional activity related to animal care and agricultural work, etc.); 3) by prophylactic measures (keeping in order water supply and sewage facilities, public health education, immunoprophylaxis, deratization/disinsectization measures, and so on).

Options that the new analytical blocks possess give them the following advantages:

convenient work with the groups of diseases interesting for a subject-matter specialist, formation of various samples from the database for a subject/region/ age group;
easy preparation of analytical materials, analysis of infectious morbidity in compliance with the current reporting forms (generation of reports and papers);
making additional retrospective epidemiological analysis (analysis of trends in epidemic processes);
analysis of groups of infections with different morbidity levels (a group analysis may appear most informative
and statistically reliable on small territories with a small population);
indirect evaluation of preventive and anti-epidemic activities (analysis of group measurement by the riskfactors, preventive measures, and epidemiological monitoring);
application of the analytical approach developed in the “Epidemiological Atlas of Russia” due to its versatility;
detailing or changing the existing groups and addition of new ones considering the interests of health authorities/organizations and Rospotrebnadzor;
improving the quality of epidemiological analysis and somatic morbidity monitoring;
close interaction with the user through the interactive elements;
standardization of the epidemiological analysis.

Clustering infections by a common feature, enhancing them with additional characteristics may serve as a basis for determining quantitative criteria of a set of infectious diseases for timely detection of changes in the epidemic situation in the territory under consideration.

## Conclusion

The developed analytical modules “Comparative analysis of morbidity by the groups of nosologies” and “Dynamic analysis of morbidity by the groups of nosologies” integrated into the geoinformation software complex “Epidemiological Atlas of Russia. Territory of the Federal District” are created to expand the analytical capabilities of this complex. They possess high significance in optimizing and improving the quality of the epidemiological monitoring, operative and retrospective epidemiological analysis on current infectious and parasitic diseases both in a separate district subject and the country as a whole; they also have the potential for improvement of analytical methods and technologies. Creation of the block for automatic generation of epidemiological reports/ bulletins (the standard of epidemiological analysis); determination of quantitative criteria of a set of infectious diseases; application of artificial neural networks in the epidemiological analysis based on the additional features of infectious diseases are the most important and promising areas of further work. The analytical modules meet the requirements of the state policy on implementation of state-of-the-art technologies into practical work. Today, there are no analogs similar to the new analytical modules.
